# Meet up‐and‐coming analytical scientists – Bharath Kumar Raghuraman

**DOI:** 10.1002/ansa.202200047

**Published:** 2023-01-01

**Authors:** Bharath Kumar Raghuraman

**Affiliations:** ^1^ Evosep Biosystems Odense Denmark; ^2^ Biomarker Laboratory Odense University Hospital Odense Denmark

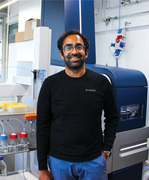

Analytical sciences are among the most dynamically developing fields and have been inherently integrated into many various scientific disciplines. At the same time, early career researchers (ECRs) are among those whose contribution to this dynamic growth cannot be simply overestimated. Hence, in this special issue “From one Early Career Researcher to the next”, we are presenting a series of editorials with Q&A from five emerging scientists of different analytical fields including omics, environmental, and data sciences. Importantly, all our guests boast not only scientific excellence and high‐quality research but also the substantial international experience gained during their Ph.D. or postdoctoral training. For this editorial, we are presenting Dr. Bharath Kumar Raghuraman.

Dr. Bharath Kumar Raghuraman comes from Chennai, a coastal city in the Southern part of India. He received his Bachelor of Technology degree from Anna University, Chennai in Biotechnology and Master of Science in Life Science and Technology from the Delft University of Technology, The Netherlands. He then pursued his doctoral studies in the lab of Dr. Andrej Shevchenko at the Max Planck Institute of Molecular Cell Biology and Genetics in Germany, where he developed mass spectrometry (MS)‐based proteomics approaches to quantify the molar concentrations of metabolic enzymes in *Caenorhabditis elegans* and understand how they act as metabolic rheostats during development. Currently, he is working as a Project scientist/Industrial Postdoc between Evosep Biosystems which manufactures liquid chromatography (LC) systems, and Odense University Hospital in Denmark developing clinical assays.

## What is your original background?

I did my Bachelor's in Biotechnology in India. My bachelor's was an interesting amalgamation of basic engineering concepts and biology, giving a holistic introduction to various subjects. I then went to Delft University of technology in the Netherlands to pursue my Master's in life science and technology. I specialized in something called ‘Cell Factory’ which included concepts from molecular biology and metabolic engineerings like genetic engineering, industrial microbiology, and systems biology. During my master's I got into MS and decided to pursue my Ph.D. with Andrej Shevchenko at the Max Planck Institute of Molecular Cell Biology and Genetics developing methods for MS‐based quantitative proteomics to answer various biological questions.

## What is your current research focus?

I work in Denmark as an industrial postdoc jointly between a company called Evosep, which provides LC solutions, and the Odense University Hospital developing LC‐MS‐based targeted clinical assays. As a part of an innovation fund project, I am currently developing a high‐throughput fully automated SRM assay for quantification of glycated albumin for diabetes diagnosis using the high throughput standardized methods developed in collaboration with Evosep, I am also involved in other method development efforts at Evosep to push the boundaries in high‐throughput quantitative proteomics.

## What is your biggest motivation to work in analytical science?

I got introduced to analytical science during my bachelor's. I still vividly remember my first TLC plate, where I was told to resolve amino acids, and how curious and excited I was to do this experiment. During my master's, I enrolled in an MS‐based proteomics course, which was love at first sight. My master's thesis mentors Dr. Peter Verhaert and Dr. Martijn Pinkse told me that I caught the ‘Mass spectrometry virus’ as I walked to them right after the first lecture series and asked if I can work with them on MS‐based proteomics for my master's thesis. I started working with MS when I was part of a team representing the university in international genetically engineered machines. We genetically engineered the *E. coli* to express antimicrobial peptides and used MS to verify whether our antimicrobial peptides were successfully expressed. I then continued my master's thesis in the same lab where I was instrumental in developing top‐down proteomics approaches to analyze human aqueous humour from donor eyes and established recommendations for assessing the quality of the cornea to increase the success of transplantation. In this project, I worked closely with Netherlands Institute for Innovative Ocular Surgery (NIIOS).

## Of all your research projects, which one was your favourite and why?

Without a doubt my Ph.D. research. Since my Ph.D. mentor is a strong proponent of reporting absolute molar quantities instead of fold changes, I focused on quantifying molar amounts of metabolic enzymes using roundworm, *C. Elegans* as the model system. The trajectory of the project fed my creativity, which helped us to interpret the mere molar amounts numbers efficiently and use them to predict growth outcomes. This journey also led us to develop a global proteome absolute quantification method called ‘Median based absolute quantification’ where we developed a sample agnostic protein standard that was used as an internal calibrant that would give us molar amounts of 1000's proteins in on go. The 4 years of my Ph.D. helped me to grow personally and professionally, so it is and will always be closer to my heart.

## What was your motivation for choosing postdoctoral training?

After my Ph.D., I wanted to take the traditional route of post‐doctoral training in an academic environment. The prime motivation for me was to hone my skills in MS‐based omics and use them to develop new workflows. So, I used this as my criteria to screen for positions and that's how I ended up getting this unique opportunity of working closely with the company Evosep and the university hospital to develop actionable clinically relevant assays.

## What was your biggest (if any) culture shock experience in the country of your postdoc?

Since I moved out of India in 2012 for my Master's, I adapted very well to the European culture and their way of living. The work‐life balance in Denmark or any Scandinavian country for that matter is given prime importance and witnessing it firsthand is indeed an experience in itself. A 37.5‐h work week and flexible work hours do provide me with sufficient time to pursue my hobbies and travel.

## In your scientific career, what was the best or worst advice you ever heard from anyone?

In my scientific career, I was very lucky to have great mentors who were always supportive and gave critical input which helped me grow as a better thinker. The best advice I had was “pursue your passion no matter what and always stand up for yourself”. The worst advice I had were from my acquaintances in India who said, I will be jobless if I pursue Biotechnology as it doesn't hold a better future. Some even went to the extent of saying, it will be difficult for me to have a family and settle down.

## What advice would you give to someone looking for a postdoc position now?

My only advice would be to also look beyond academia without compromising much on scientific interests. The pandemic though negatively impacted the world, gave a much‐needed boost and attention to analytical sciences. This pushed various pharmaceutical companies to establish or strengthen their analytical sciences team. So, in short, my advice will be ‘You can also conduct cutting‐edge research outside academia, so have an open mind’.

## What is your favourite non‐scientific activity?

I am a trained Indian classical musician and learning western music so, I always like to sing or have occasional jam sessions with my jazz music teacher. I also love cooking and gardening as both of them are quite therapeutic for me. I got interested in specialty coffee during the pandemic and started exploring specialty coffee shops in various cities with a plan to open one myself sometime.

## Who (three people but not scientists!) would you invite to a dream dinner party?

Hmm… That's very tough for me but, if I had to choose it would be: (1) (Late) Dr. A.P.J. Abdul Kalam, President of India; (2) Dr. Jordon Peterson, clinical psychologist; and (3) J.K. Rowling, author. All these personalities helped shape my views on various aspects of life and would be great to have a one‐on‐one masterclass with them over dinner.

## CONFLICT OF INTEREST

The author declares that he has no conflict of interest.

